# Effect of post-processing on the surface, optical, mechanical, and dimensional properties of 3D-printed orthodontic clear retainers

**DOI:** 10.1007/s00784-024-06120-4

**Published:** 2025-01-06

**Authors:** Siew Peng Neoh, Anak Khantachawana, Peerapong Santiwong, Rochaya Chintavalakorn, Toemsak Srikhirin

**Affiliations:** 1 Department of Orthodontics, Faculty of Dentistry, Mahidol University, 6 Yothi Alley, Phayathai, Bangkok, 10400 Thailand; 2 Department of Mechanical Engineering, Faculty of Engineering, King Mongkut’s University of Technology Thonburi, 126 Pracha Uthit Road, Bang Mot, Thung Khru, Bangkok, 10140 Thailand; 3 School of Materials Science and Innovation, Faculty of Science, Mahidol University, Phuttamonthon 4 Road, Salaya, Phuttamonthon District, Nakhon Pathom, 73170 Thailand

**Keywords:** 3D printing, Clear retainers, Post-processing, Centrifugation, Coating

## Abstract

**Objectives:**

To address the high surface roughness and poor optical properties of three-dimensional (3D) printed orthodontic clear retainers, an alternative post-processing protocol was investigated with the goal of achieving improved surface, optical, and mechanical properties while preserving dimensional accuracy.

**Materials and methods:**

Samples were prepared from two biocompatible methacrylate-based 3D-printing resins (Formlabs Dental LT Clear V2, NextDent OrthoFlex) and one thermoplastic material (Duran). For the 3D-printed resins, one group was post-processed by rinsing in isopropyl alcohol, while another group was centrifuged before post-curing in glycerine. Three different testing conditions were used: dry, wet (24-h water immersion), and aged (thermocycling for 10,000 cycles). Surface characteristics were evaluated qualitatively and quantitatively. Optical properties were assessed for transparency and colour stability, while mechanical properties were elicited from tensile and microhardness tests. Water sorption and solubility were calculated. Samples mounted on a dental model were scanned by micro-computed tomography to measure thickness and gap width.

**Results:**

3D-printed samples post-processed by centrifugation showed significantly decreased surface roughness and improved visible light transmission, colour stability, tensile strength, and hardness. The centrifuged samples showed significantly increased thickness, while designing an offset equal to this thickness improved the adaptation.

**Conclusions:**

Post-processing by centrifugation produces surface coating that enhances the surface and optical properties of the 3D-printed orthodontic retainers, while curing in an oxygen-free environment improves their mechanical properties. Design modifications may be necessary for this protocol to ensure proper adaptation to the dentition.

**Clinical relevance:**

Proper design and post-processing protocols are necessary to achieve the desired properties of orthodontic clear retainers.

## Introduction

The use of additive manufacturing or three-dimensional (3D) printing technology has increasingly become more popular for the fabrication of dental appliances. The 3D-printing workflow has its benefits by decreasing the reliance on manpower and production time, reducing physical storage, and opening up the possibilities in appliance design [1–3]. In orthodontics, 3D-printing has been used primarily for study models and various orthodontic appliances. In order to replace conventional fabrication techniques, the 3D-printing workflow should demonstrate equal if not more effectiveness in the end product.

Orthodontic clear retainers that were introduced since 1971 have gained in appeal among practitioners and patients because of their improved aesthetics, comfort, speech, and relative ease of fabrication [4, 5]. However, the thermoforming process produces appliances of non-uniform thickness and fit, and the dental models which are discarded after use generates a substantial amount of waste. 3D-printing has the potential to reduce this unnecessary waste by direct fabrication of the custom-designed retainers without needing an intermediary model [1, 6–8].

The additive layer-by-layer deposition in 3D-printing is both the strength and limitation of the technology. The former enables the production of complex objects, while the latter may compromise certain properties due to its anisotropic nature [9]. For clear retainers, it is vital that the materials have good surface, optical, and mechanical properties to encourage patient compliance with full-time wear for at least six months to reduce the rate of relapse after the completion of orthodontic treatment [10, 11].

The properties of 3D-printed appliances are dependent upon the 3D-printing technology, material composition, layer thickness, print orientation, and post-processing protocols [12]. The most suitable technology related to the 3D-printing of polymeric materials for clear retainers is vat photopolymerization. Depending on the nature of the light source, this technology is divided into several subtypes, which are stereolithography (SLA), digital light processing (DLP), and liquid crystal display (LCD) [13, 14]. The raw material essentially comprises liquid oligomers and photoinitiators which are sensitive to wavelengths in the range of 385 to 405 nm of an adequate intensity and duration. Immediately after 3D-printing, the objects are only partially polymerized and are covered in uncured resin. Additional post-processing steps are required to enhance the degree of polymerization and achieve the desired properties [15, 16].

The layer of unpolymerized resin is usually rinsed off in an appropriate solvent such as isopropyl alcohol (IPA), but this has been shown to negatively affect the surface and optical properties of the appliance [17]. Low surface roughness is important for intraoral dental appliances to ensure high strength, colour stability, patient comfort, and transparency in the case of clear appliances, as well as to reduce biofilm adhesion [9, 18, 19]. Previous efforts on surface treatment of 3D-printed denture base materials have demonstrated the benefits of mechanical polishing or protective coating. Out of the two, it is impractical to polish 3D-printed clear retainers due to their complex morphology that conforms to the anatomy of the dentition and gingiva. Several recent studies on 3D-printed orthodontic clear aligners have suggested centrifugation to leave a thin surface coat of resin before the final post-curing step [17, 20].

The post-curing environment also affects the mechanical properties of 3D-printed appliances. Curing in an oxygen-free environment would be beneficial as oxygen would inhibit the ultraviolet (UV)-initiated free-radical polymerization, resulting in a low degree of conversion (DC) [21] and the formation of partially cured ‘tacky’ surfaces [22]. An oxygen-free atmosphere can be achieved by using a vacuum or inert gases such as nitrogen, helium, and carbon dioxide [23, 24], or by using a physical oxygen diffusion barrier such as glycerine [25, 26].

At the time of writing, no study has yet been conducted on how different post-processing protocols would affect the properties of 3D-printed clear retainers. Therefore, the objective of this study was to compare the surface, optical, and mechanical properties between IPA rinsing with centrifugation of 3D-printed clear retainers, while ensuring their dimensional accuracy.

## Materials and methods

### Sample preparation

**Fig. 1:**
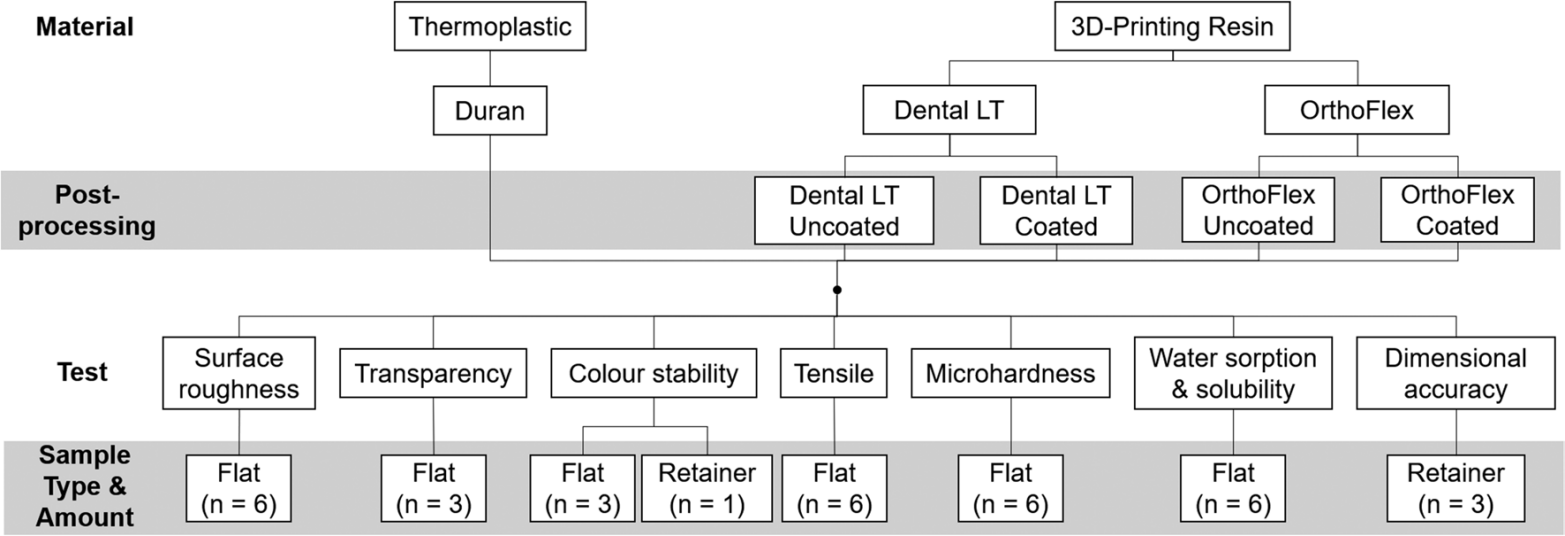
Flowchart of the experimental design

A summary of the different groups and tests performed in this study is depicted in Fig. 1. Two 3D-printing resins were used: Dental LT Clear V2 (Dental LT; Formlabs Inc., Mass., USA) [27] and OrthoFlex (3D Systems, Vertex-Dental, Soesterberg, Netherlands) [28]. Each resin was further divided according to the post-processing protocol, giving the following four groups: Dental LT Uncoated, Dental LT Coated, OrthoFlex Uncoated, and OrthoFlex Coated. The control group consisted of a polyethylene terephthalate glycol (PET-G) thermoplastic material (Duran 0.75 mm, Scheu Dental GmbH, Iserlohn, Germany) [29].

All flat 3D-printed samples were digitally constructed in a 3D design software (3D Builder, Microsoft Corp., WA, USA), with a thickness of 0.75 mm and dimensions as required by each testing apparatus. For the retainer samples, a maxillary dental model was first scanned using an intraoral scanner (TRIOS3, 3Shape Dental Systems, Copenhagen, Denmark) and exported to a 3D software (Appliance Designer, 3Shape Dental Systems, Copenhagen, Denmark), where retainers of 0.75 mm thickness were designed on the digital model. All files were saved in Standard Tessellation Language (.STL) format and 3D-printed at 0.100 mm layer thickness at a 45° orientation. After 3D-printing, the supports were removed before post-processing. Details of the composition, fabrication, and post-processing methods for each 3D-printing material are summarized in Table 1.

**Table 1:** Post-processing conditions for each 3D-printing resin

3D-printing resin	Composition	3D-printer	Post-processing	Sample type	Resin removal	Post-curing
Dental LT Clear V2 (Formlabs Inc., Mass., USA) [27]	Bisphenol-A dimethacrylate, methacrylate monomer, urethane dimethacrylate	Form 3B (Formlabs Inc., Mass., USA)	Uncoated	Flat	Rinsed in 99% isopropyl alcohol for 20 min	Post-cured at 405 nm for 60 min at 60 °C in air
				Retainer		
			Coated	Flat	Excess uncured resin gently wiped off	Post-cured at 405 nm for 60 min at 60 °C, submerged in glycerine
				Retainer	Centrifuged at 330 rpm for 10 min	
NextDent OrthoFlex (3D Systems, Vertex-Dental, Soesterberg, Netherlands) [28]	Methacrylate ester monomer, 2-phenoxyethyl acrylate	Photon Mono X (Anycubic, Shenzhen, China)	Uncoated	Flat	Rinsed in 99% isopropyl alcohol for 20 min	Post-cured at 405 nm for 20 min at 70 °C in air
				Retainer		
			Coated	Flat	Excess uncured resin gently wiped off	Post-cured at 405 nm for 20 min at 70 °C, submerged in glycerine
				Retainer	Centrifuged at 330 rpm for 10 min	

Samples for the thermoplastic material were prepared by first designing templates of the required dimensions in the same software and 3D-printed using photopolymer resin, Dental Model V2 (Formlabs Inc., Mass., USA) using the same SLA 3D printer (Form 3B, Formlabs Inc., Mass., USA). The 3D-printed templates were rinsed in 99% IPA (Form Wash, Formlabs Inc., Mass., USA) and allowed to air-dry before post-curing at 60 °C for 20 min (Form Cure, Formlabs Inc. Mass., USA). The thermoplastic sheets were then thermoformed over the 3D-printed templates in a thermoforming machine (Biostar, Scheu Dental GmbH, Iserlohn, Germany) [29] according to the manufacturer’s instructions, before being cut out to the required dimensions for each testing procedure.

Tests for surface and mechanical properties were performed on samples subjected to three conditions to simulate the different service conditions of orthodontic retainers:

- Dry: Tests were conducted on samples ‘as is’ at ambient room temperature of 25 °C and 50% humidity
- Wet: Tests were conducted on samples after immersion in distilled water at 37 °C for 24 h
- Aged: Tests were conducted after samples were subjected to thermocycling for 10,000 cycles, where one cycle consisted of 25 s each in 5 °C and 55 °C water baths, with 2 s dripping time (equivalent to one-year service condition [30, 31])

### Surface properties

Qualitative surface assessment was done by observing samples of each group under Scanning Electron Microscopy (SEM) (JSM-6610LV, JEOL Ltd., Tokyo, Japan) with a beam voltage of 15 kV, working distance of 18 mm, and at a magnification of 500x. Before SEM observation, the sample surfaces were coated with gold by vacuum evaporation using an ion sputter coater (SC7620, Quorum Technologies Inc., United Kingdom). Quantitative surface assessment was performed by 3D laser confocal microscopy (Olympus OLS5100, Olympus Corp., Japan). Six samples measuring 10 × 10 mm were prepared for each group. Surface scans were done under 50 × magnification for an area of 257.5 × 258.0 μm. The same region of the same sample for dry, wet, and aged conditions was measured for the following parameters, as defined by the International Organization for Standardization (ISO) 25,178–2:2021: 1) Arithmetical mean height, Sa (μm) which is the difference in height of each point compared to the arithmetical mean of the surface and 2) Maximum height, Sz (μm) which is measured from the highest peak to the deepest valley within the defined area [32].

### Optical properties

Light transmittance was measured in the wavelength range of 300 to 800 nm using an ultraviolet–visible (UV–Vis) light spectrophotometer (Cary 60, Agilent Technologies, Santa Clara, CA, USA). Three samples measuring 24 × 24 mm per group were analysed.

For colour stability tests, two forms of samples were prepared. The first form were square-shaped samples of 15 × 15 mm. The second form were designed as orthodontic clear retainers, but only covering up to the maxillary first premolar areas. Four staining solutions were prepared as described in Table 2, with the control group being distilled water. Three square-shaped and one retainer sample per group were immersed in each solution and placed in an incubator at 37 °C. The solutions were refreshed every 24 h.

**Table 2:** Preparation of staining solutions for tests of colour stability

Staining solution	Brand	Preparation
Black coffee	Nescafe Red Cup (Nestle, Thailand)	2 g coffee powder diluted in 100 mL boiling distilled water
Black tea	Yellow Label Tea (Lipton, Thailand)	1 teabag immersed in 100 mL boiling distilled water
Red wine	Shiraz red wine (Paul Masson, Australia)	Undiluted, as is
Curry	Namya Curry Paste (Sam Phao Tong, Thailand)	20 g curry paste diluted in 100 mL boiling distilled water

The colour parameters were measured with a colourimeter (ColorFlex 45/0, Hunterlab, VA, USA) before staining (T0) and after periods of 24 h (T1) and 288 h (T2), as it has been suggested that immersion for 24 h simulates consumption of one month [33, 34]. The colour changes were characterised according to the Commission Internationale de I’Eclairage L*a*b* colour system (CIE L*a*b*). All samples were washed in an ultrasonic cleaner for 5 min and blotted dry with paper towels before the colour measurements. The total colour changes (ΔΕ*) between T1-T0 and T2-T0 were calculated according to ISO 28642:2016 [35] using the following equation:

$$\Delta {E}^{*}={[{\left(\Delta {L}^{*}\right)}^{2}+{\left(\Delta {a}^{*}\right)}^{2}+{\left(\Delta b\right)}^{2}]}^\frac{1}{2}$$

The National Bureau of Standards (NBS) system was used to describe the levels of visually perceptible colour change to relate the colour changes to a clinical standard (Table 3). The ΔE* values were converted into NBS units with the equation NBS = ΔE* × 0.92 [36, 37].

**Table 3:** Description of National Bureau of Standards (NBS) units

National Bureau of Standards (NBS) units	Description of colour change
0.0 – 0.5	Trace: extremely slight change
0.5 – 1.5	Slight: slight change
1.5 – 3.0	Noticeable: perceivable
3.0 – 6.0	Appreciable: marked change
6.0 – 12.0	Much: extremely marked change
12.0 or more	Very much: change to another colour

For the retainer samples, they were fitted on a maxillary dental model with natural tooth and gingival shades to simulate the appearance of the retainers in actual use. Colour photographs of the anterior region were taken with a digital single-lens reflex camera (650D, Canon Inc., Japan) fitted with a macro lens (EF-S 600 mm f2.8 Macro USM, Canon Inc., Japan) and macro ring-flash system (MR14EX II, Canon Inc., Japan). The same position of the camera and dental model set-up was used for all photographs at all time points, taken in the same room under ambient lighting.

### Mechanical properties

Tensile tests were carried out on 45 mm × 15 mm samples for each group (*n* = 6), with a gauge length of 15 mm and gauge width of 5 mm. A universal testing machine (Instron 5577, Buckinghamshire, United Kingdom) with a 1 kN load cell at a crosshead speed of 5 mm/min was used for testing.

Another six samples measuring 10 × 10 mm were prepared for each group and tested for Vickers hardness values using a microhardness tester (FM-ARS 9000, Future-Tech Corp., Kanagawa, Japan), using a force of 300 g for 15 s.

### Water sorption and water solubility

To measure water sorption and water solubility, six disc-shaped samples of 15 mm diameter were prepared for each group according to ISO 4049:2019 [38]. All samples were stored in the first desiccator at 37 °C for 22 h, then transferred to a second desiccator at 23 °C for 2 h before being weighed using a digital analytical scale (AP225WD, Shimadzu Corp., Japan). This process was repeated until a constant mass, *m*_*1*_ was obtained. The volume, *V* of each sample was calculated from measuring the sample diameter and thickness with a digital vernier calliper (Mitutoyo Corp., Japan). The samples were then immersed in distilled water at 37 °C for 7 days before being weighed again, recorded as *m*_*2*_. The drying cycles using the two desiccators were repeated until a constant mass, *m*_*3*_ was achieved. The water sorption and water solubility values were calculated as follows:

$$\begin{array}{c}Water sorption=\frac{{m}_{2}-{m}_{3}}{V}\\ Water solubility=\frac{{m}_{1}-{m}_{3}}{V}\end{array}$$

### Dimensional properties

For dimensional accuracy testing of each 3D-printing material, three retainer designs with different offsets of 0 mm, 0.1 mm, and 0.2 mm were constructed in the 3D software and prepared as described earlier. The retainer samples were fitted onto the same maxillary dental model which was used to digitally design the samples. 3D images of the samples were generated using a micro-CT scanner (SKYSCAN 1173, Bruker, Belgium). The object and camera distances to the source were 256.9 and 364.0 mm, respectively. Scanning was conducted using a flat panel detector with 50 µm pixel size. The beam voltage was set at 80 kV, amperage at 180 μA, and the voxel size was 34.86 μm. The total scanning time was under 60 min per sample. The resulting data were used to create a digital volumetric reconstruction in the NRecon software (Version 1.7.5.1, Bruker, Belgium).

The teeth chosen for dimensional analysis were the right maxillary central incisor, canine, and first permanent molar. The landmarks used for measurement were the mid-buccal surface, mid-palatal surface, and incisal or cusp tips of each tooth, giving a total of 10 landmarks for each sample.

Using imaging software (Dragonfly, Object Research Systems Corp., Montreal, Canada), a cross-sectional plane through the middle of each tooth was constructed perpendicular to the plane joining the mesial and distal tooth surfaces. At each landmark, the ‘Ruler’ tool in the software was used to trace a line perpendicular to the surface tangent to measure the thickness of the retainer and the width of the gap between the inner surface of the retainer with the tooth surface.

### Statistical analysis

Statistical analysis was performed in the Statistical Package for Social Sciences (Version 18, IBM Corp., Armonk, NY). Comparison of means between the different materials and the different test conditions were carried out using one-way ANOVA and post-hoc Tukey tests, with the level of significance set at *p* < 0.05.

## Results

### Surface properties

**Fig. 2:**
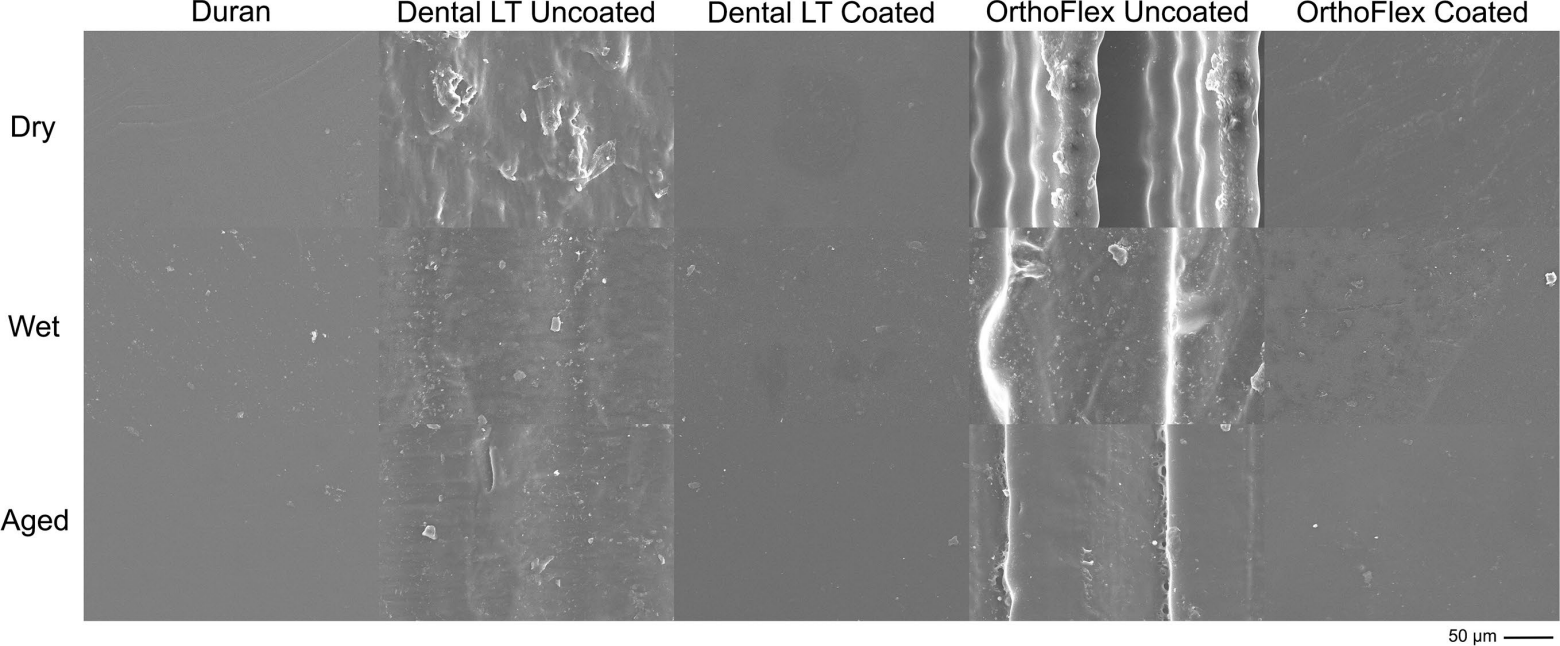
SEM images of the surface of each group subjected to three different conditions. (× 500 magnification)

From the SEM images (Fig. 2), the surface characteristics of each group can be clearly appreciated. In the dry state, the thermoplastic Duran shows an almost uniform surface with minimal irregularities. Dental LT Uncoated presented with a rough surface and relatively large deposits, while OrthoFlex Uncoated presented with a gradient of irregular, undulating layers. These characteristic surfaces reflect the nature of the individual 3D-printing technologies. Dental LT Coated and OrthoFlex Coated showed almost homogenous surfaces, with slightly more irregularities seen in the latter group. Both Coated groups were strikingly distinct from their Uncoated counterparts, and not unlike the control group Duran.

Water immersion decreased the surface unevenness of Dental LT and OrthoFlex Uncoated, with the multi-layer structure diminished to leave only one main layer. A decrease in the amount and size of surface deposits was also observed. In Duran and the Coated groups, a slight increase in surface impurities and irregularities was seen after water immersion, but these disappeared after ageing. The same effects from ageing were seen in the Uncoated 3D-printing materials.

**Fig. 3:**
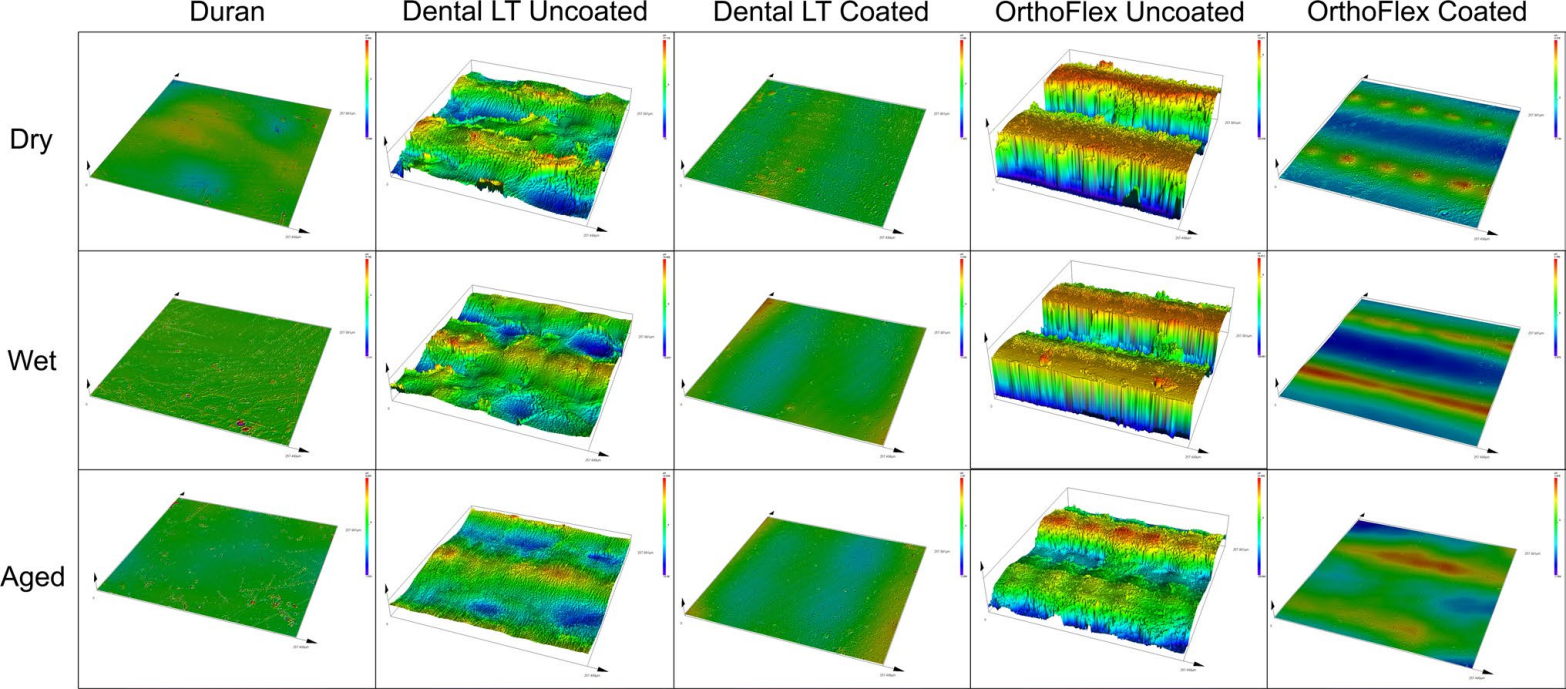
Colour surface mapping of 3D laser microscopic images. Please note the differences on the y-axis scale. (× 50 magnification)

The 3D variations in the surface morphology between groups are shown in Fig. 3. Dental LT Uncoated displayed irregular undulations where a multi-layer structure can be faintly detected, while clear demarcations of multi-layers with flat plateaus and valleys were apparent in OrthoFlex Uncoated. Dental LT Coated more closely resembled the control group Duran with an almost even surface morphology, while OrthoFlex Coated still showed the presence of high and low layers, but these were markedly much reduced compared to the Uncoated group. Similar to the SEM findings, water immersion and ageing decreased the height discrepancies of the surface ripples in all groups.

**Fig. 4:**
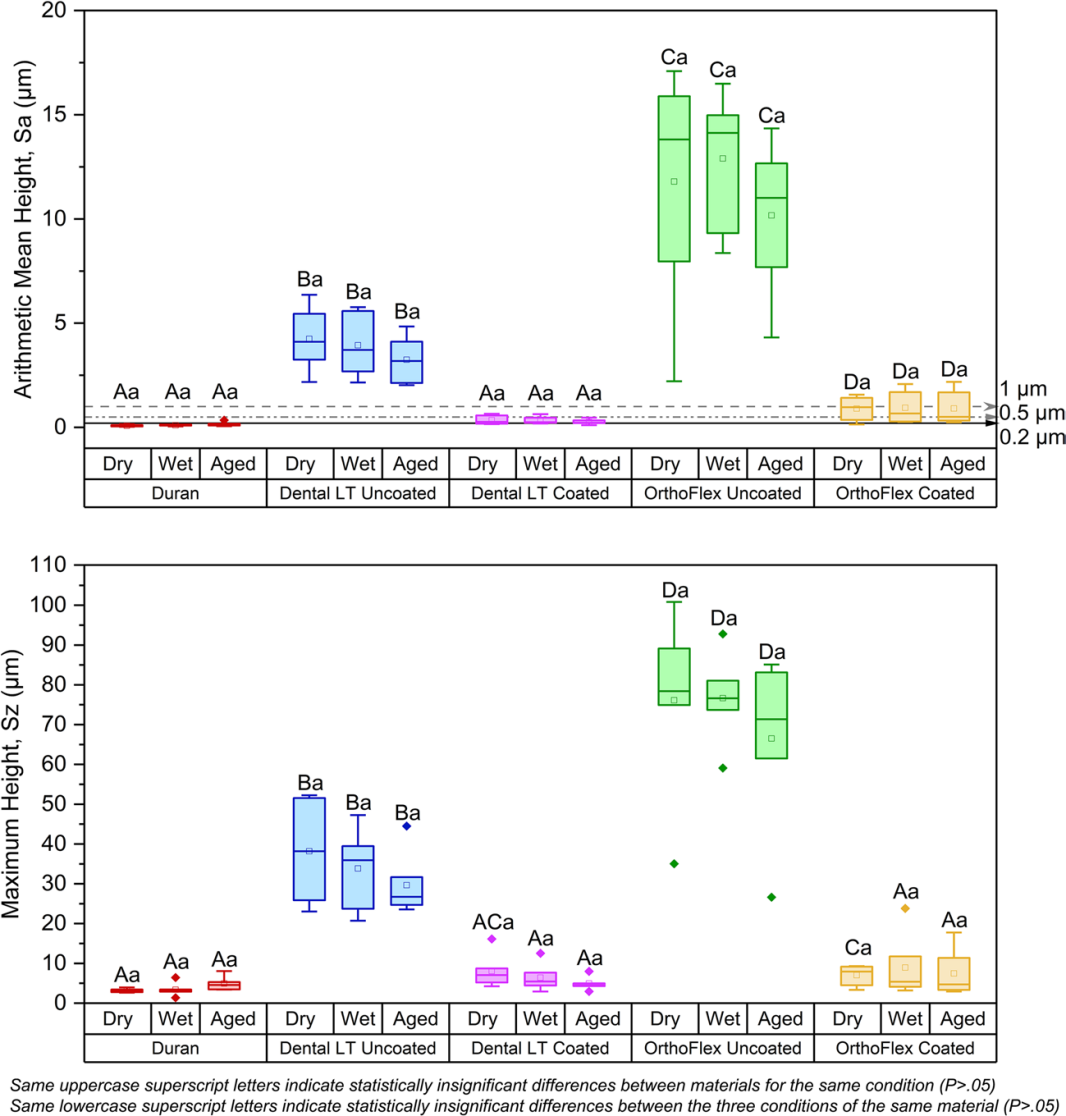
Surface roughness parameters (Sa and Sz) of each group under three different conditions

The quantitative measurements of surface roughness further reinforce the observations from the SEM and 3D confocal images. (Fig. 4) In all conditions, the thermoplastic Duran had the lowest mean Sa and Sz values below 0.2 μm and 5 μm respectively, while the highest were seen in OrthoFlex Uncoated with mean Sa above 10 μm and mean Sz above 65 μm. The coated 3D-printing resins showed statistically significant decreases in Sa and Sz of more than 90% (*p* < 0.05), with lower values seen in Dental LT compared to OrthoFlex Coated but these were not statistically significant (*p* > 0.05). The Sa and Sz values of Dental LT and OrthoFlex Coated in almost all conditions were also statistically similar to the control group, Duran (*p* > 0.05), the only exceptions being the Sa of aged OrthoFlex Coated and Sz of dry Dental T Coated. Statistical analysis confirmed that both surface roughness parameters of all groups were not significantly affected by the dry, wet, or aged conditions (*p* > 0.05).

### Optical properties

**Fig. 5:**
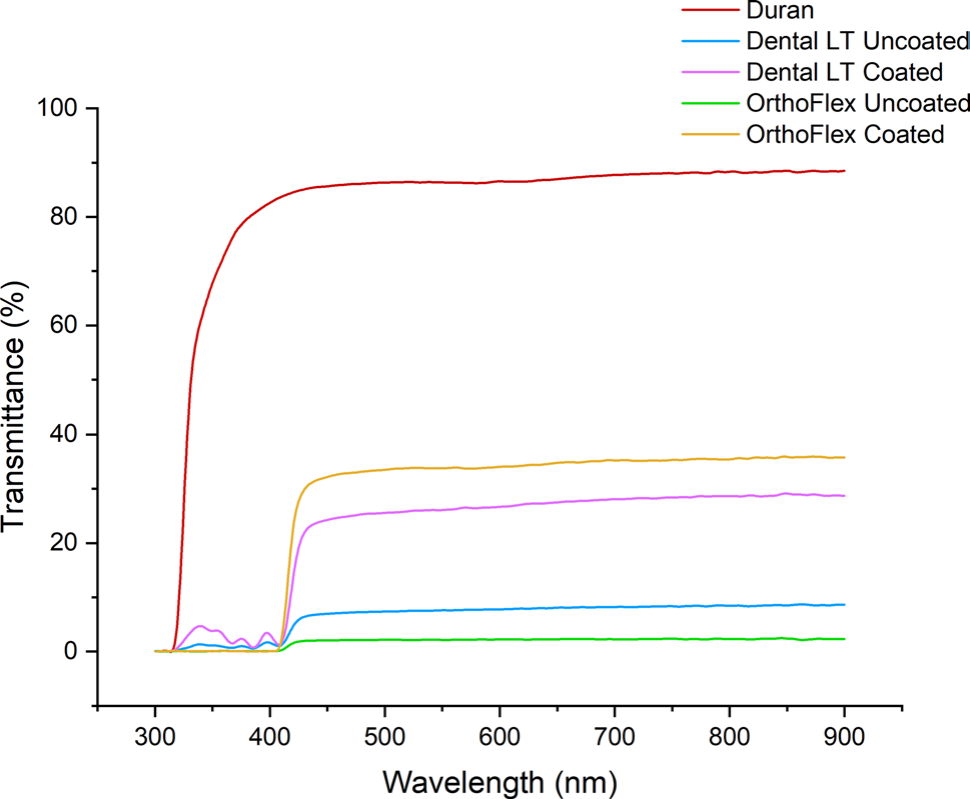
Combined UV–vis transmittance spectra of each group

The light transmittance of the thermoplastic Duran was highest at above 80%, indicating a high degree of transparency. Within the range of visible light from 400 to 700 nm, the transmittance of Dental LT Uncoated was about 5–10%, while OrthoFlex Uncoated was the lowest at less than 5%. In this same range, the coated 3D-printing groups both had a large increase in transmittance to about 75%, but still slightly lower than Duran. (Fig. 5).

**Fig. 6:**
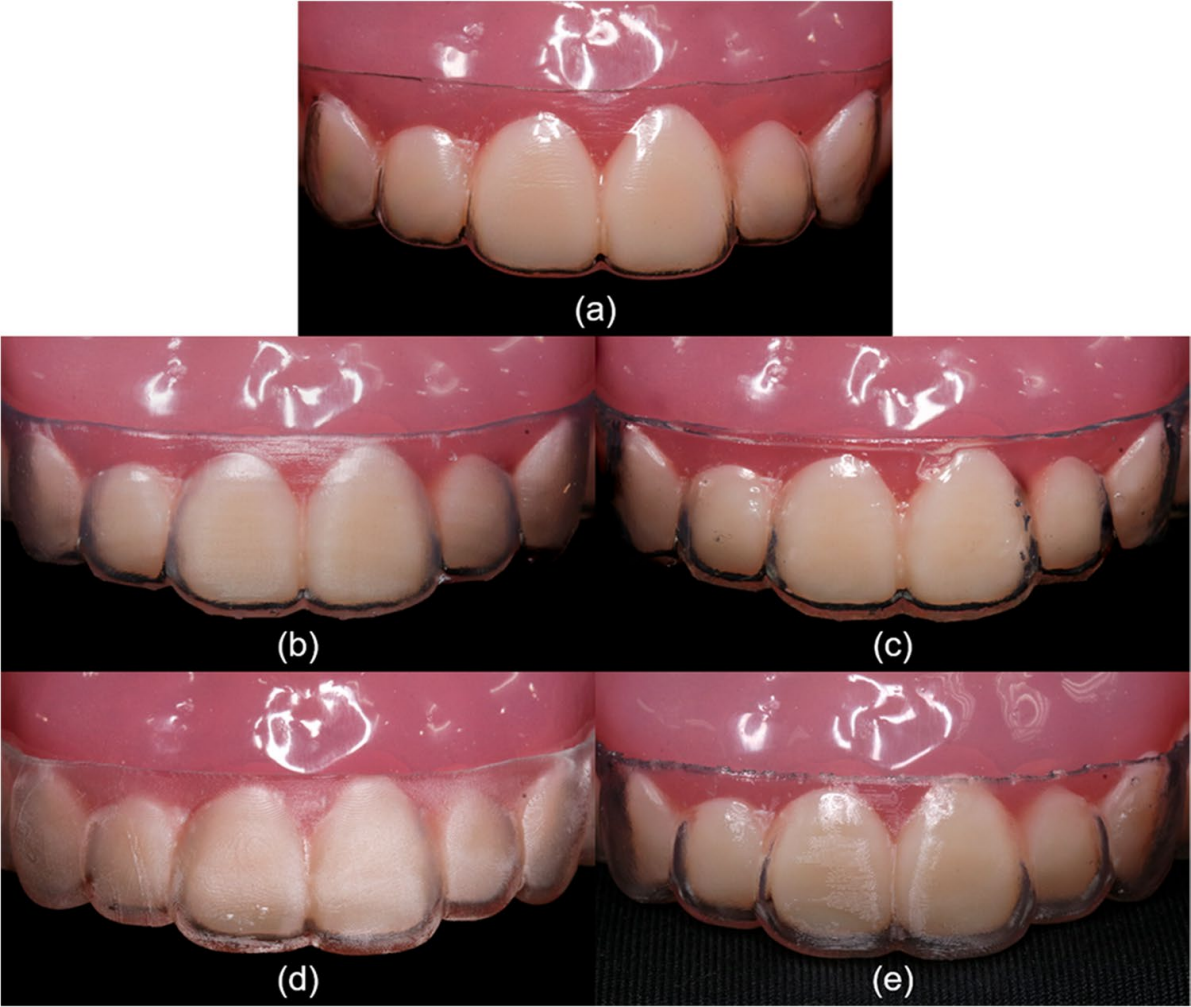
Photographs of clear retainer samples from each group mounted on a typodont. **a** Duran; **b** Dental LT Uncoated; **c** Dental LT Coated; **d** OrthoFlex Uncoated; **e** OrthoFlex Coated

From the retainer samples mounted on the maxillary model, Duran showed the most even, glossy, and transparent surface. (Fig. 6a) Both Uncoated groups appeared translucent and non-glossy, more so in the OrthoFlex material. (Fig. 6b and d) The Coated groups showed improved surface evenness, transparency, and gloss, especially for the Dental LT material. (Fig. 6c and e).

**Fig. 7:**
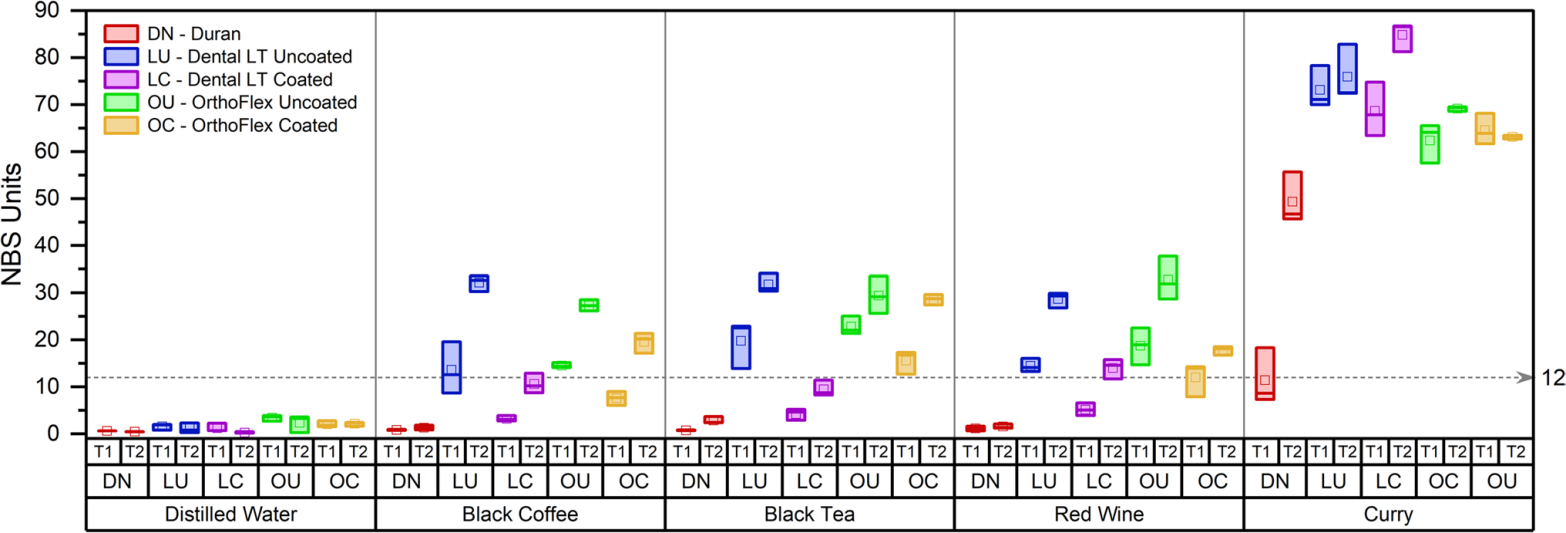
Quantitative colour changes of each material after immersion in different staining solutions for T1 (24 h) and T2 (12 days)

Immersion in distilled water up to 12 days produced minimal colour changes in all groups, with OrthoFlex Uncoated having the highest values of ‘Noticeable’ change at around 3 NBS units. The thermoplastic Duran was least affected by immersion into all staining solutions, except for curry, where colour change was ‘Very much’ even after only 24 h of immersion. All uncoated 3D-printing groups had NBS units higher than 12 for all staining solutions at T1 and T2. Dental LT Coated performed better than OrthoFlex Coated in response to black coffee, black tea, and red wine, with all T1 values being less than 5 units, but T12 values were higher around 10 to 15 units. OrthoFlex Coated appeared more susceptible to colour change, especially in black tea. All groups including Duran, however, showed the greatest colour change after immersion in curry. (Fig. 7).

**Fig. 8:**
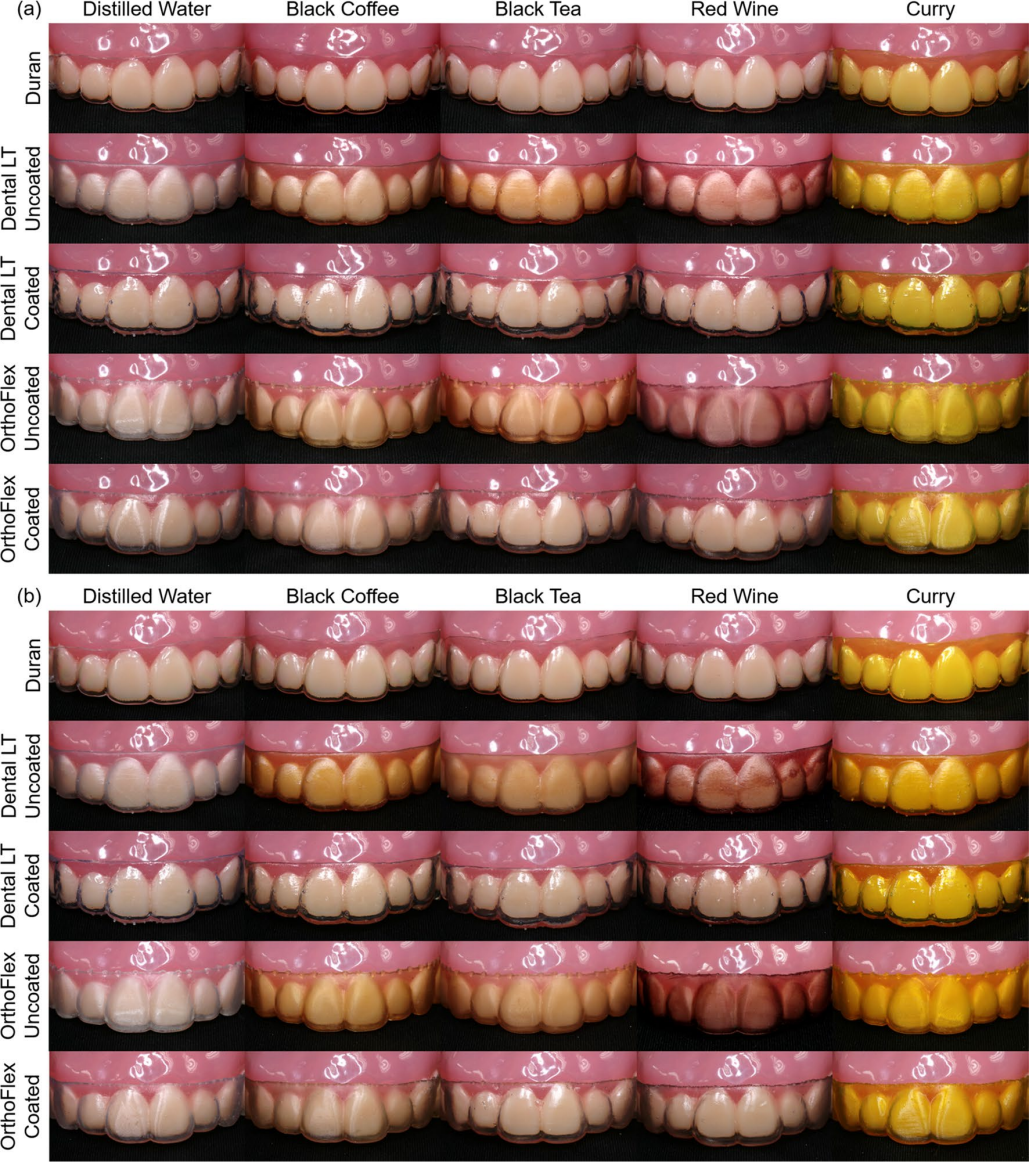
Photographs of clear retainer samples mounted on a typodont after immersion into different staining solutions for: **a** 24 h; **b** 12 days

When comparing the NBS values with visual inspection of the photographs of the retainer samples, marked colour changes were seen in Dental LT and OrthoFlex Uncoated for all staining solutions, as well as for exposure to curry irrespective of material type or duration of immersion. (Fig. 8).

### Mechanical properties

**Fig. 9:**
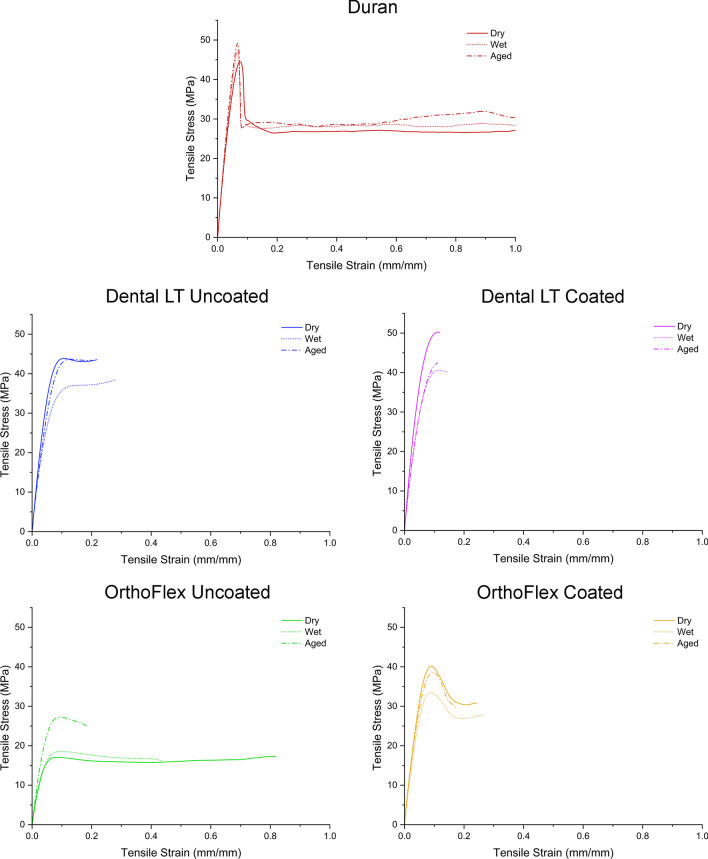
Stress–strain curves of each group in dry (solid line), wet (dotted line), and aged conditions (dot-and-dash line). All curves have been plotted on the same scales. (Please note that the strain at break is not depicted for Duran)

The highest values for all tensile parameters were consistently seen in the thermoplastic Duran. OrthoFlex Uncoated had the lowest tensile strength, elastic modulus, yield stress, and yield strain values, but the lowest strain at break was observed in Dental LT Coated. (Fig. 9, Table 4).

**Table 4:** Various tensile properties of each material in dry, wet, and aged conditions

Material	Condition	Tensile strength (MPa)		Elastic modulus (MPa)		Strain at break (mm/mm)		Yield stress (MPa)		Yield strain (mm/mm)	
		Mean	SD	Mean	SD	Mean	SD	Mean	SD	Mean	SD
Duran	Dry	43.31^Aa^	1.54	882.91^Aa^	44.84	3.22^Aa^	0.61	24.58^Aa^	2.19	0.03^Aa^	0.00
	Wet	47.66^Ab^	2.53	891.65^Aa^	75.80	3.44^Aa^	0.36	24.02^Aa^	1.45	0.03^Aa^	0.00
	Aged	52.13^Ac^	3.63	895.67^Aa^	80.35	2.29^Ab^	0.30	26.83^Aa^	4.18	0.03^Aa^	0.01
Dental LT Uncoated	Dry	46.60^Aa^	4.16	817.56^Aa^	91.79	0.23^Ba^	0.04	23.45^Aa^	2.54	0.03^Aa^	0.00
	Wet	39.36^Bb^	1.91	620.86^Bb^	51.50	0.29^Ba^	0.07	19.44^Bb^	1.15	0.03^Aa^	0.00
	Aged	43.52^Bab^	1.55	683.06^Bb^	18.42	0.21^Ba^	0.05	21.57^Bb^	0.96	0.03^Aa^	0.00
Dental LT Coated	Dry	49.63^Aa^	1.46	873.39^Aa^	53.40	0.12^Ca^	0.03	23.99^Aa^	1.29	0.03^Aa^	0.00
	Wet	39.73^Bb^	2.65	688.48^Bb^	63.08	0.14^Ca^	0.03	19.14^Bb^	2.41	0.03^Aa^	0.00
	Aged	41.31^Bb^	2.34	676.50^Bb^	44.84	0.12^Ba^	0.02	18.67^Cb^	2.31	0.03^Aa^	0.00
OrthoFlex Uncoated	Dry	18.58^Ba^	3.39	453.78^Ba^	112.01	0.79^Da^	0.17	9.36^Ba^	2.47	0.02^Ba^	0.00
	Wet	16.46^Ca^	2.71	395.87^Ca^	58.26	0.58^Bb^	0.14	8.68^Ca^	1.51	0.02^Ba^	0.00
	Aged	26.10^Cb^	0.77	572.47^Cb^	35.61	0.21^Bc^	0.01	13.86^Db^	0.67	0.03^Aa^	0.00
OrthoFlex Coated	Dry	39.76^Ca^	1.34	758.99^Aa^	46.72	0.22^Ba^	0.06	20.10^Ca^	1.73	0.03^Aa^	0.00
	Wet	33.67^Db^	0.84	635.21^Bb^	63.84	0.32^Cb^	0.09	18.11^Ba^	2.51	0.03^Aa^	0.01
	Aged	37.59^Dc^	1.23	718.17^Bab^	59.22	0.18^Ba^	0.02	18.00^Ca^	1.17	0.03^Aa^	0.00

Same uppercase superscript letters indicate statistically insignificant differences between materials for the same condition (*p* > 0.05).

Same lowercase superscript letters indicate statistically insignificant differences between the three conditions of the same material (*p* > 0.05).

Compared to the uncoated counterparts, coating did not significantly affect the tensile strength and elastic modulus of the Dental LT material (*p* > 0.05), but these were increased significantly in OrthoFlex (*p* < 0.05). The strain at break was significantly reduced in both Dental LT and OrthoFlex Coated compared to the uncoated groups (*p* < 0.05). Yield stress significantly decreased in aged Dental LT Coated, but increased in all conditions for OrthoFlex Coated and were statistically similar to Dental LT Coated. Yield strain was not affected by coating Dental LT, but improved in OrthoFlex instead.

Regarding the effect of different conditions, the tensile strength of Duran increased significantly from both water immersion and ageing, while the 3D-printed groups showed similar trends of lower tensile strength after water immersion but higher after ageing. The elastic modulus of Duran remained the same regardless of condition, but reduced after water immersion and ageing in both Dental LT Uncoated and Coated. In OrthoFlex Uncoated and Coated however, tensile strength, elastic modulus, and yield stress were the highest after ageing. The strain at break was significantly decreased in aged Duran and OrthoFlex Uncoated, but no changes were seen in the remaining groups. The yield strain of all materials was not significantly affected by water immersion and ageing.

**Fig. 10:**
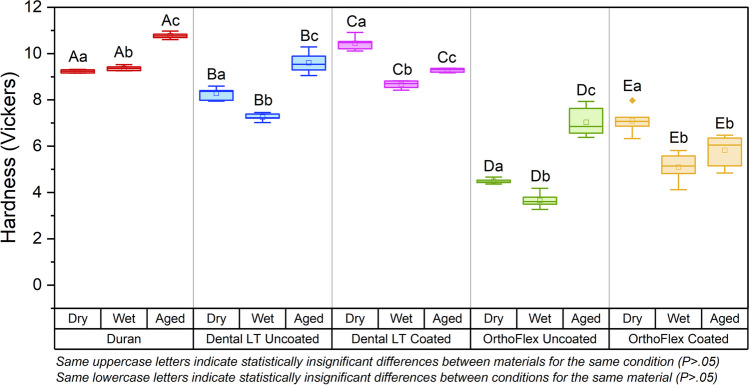
Vickers microhardness values for each material in dry, wet, and aged conditions

The surface microhardness of all groups was significantly different between the dry, wet, and aged conditions (*p* < 0.05). The only exception was for OrthoFlex Coated, where the wet and aged conditions were statistically similar (*p* > 0.05). The highest Vickers microhardness was seen in aged Duran (mean ± SD: 10.77 ± 0.13), while the lowest was in wet OrthoFlex Uncoated (mean ± SD: 3.66 ± 0.31). Water immersion decreased the hardness of all groups except Duran, while ageing reversed this effect to various extents. The coated groups of both 3D-printing materials generally showed significantly increased hardness compared to the Uncoated groups in all test conditions. (Fig. 10).

### Water sorption and water solubility

The lowest water sorption and water solubility values were seen in the thermoplastic Duran at 11.835 ± 0.714% and 0.809 ± 0.051%, respectively. Coating significantly decreased water solubility for Dental LT, but no change was seen in water sorption. Both values were significantly reduced by coating for OrthoFlex, and were statistically similar to those of Duran. (Table 5).

**Table 5:** Water sorption and water solubility values of the tested groups

Material	Water sorption (%)		Water solubility (%)	
	Mean	SD	Mean	SD
Duran	11.835^A^	0.714	0.809^A^	0.051
Dental LT Uncoated	25.839^B^	2.651	5.131^B^	2.259
Dental LT Coated	25.205^B^	1.209	3.122^C^	0.437
OrthoFlex Uncoated	13.648^AC^	0.545	6.198^B^	0.520
OrthoFlex Coated	11.996^AD^	1.893	1.232^AC^	0.144

Same uppercase superscript letters indicate statistically insignificant differences between materials (*p* > 0.05).

### Dimensional properties

**Fig. 11:**
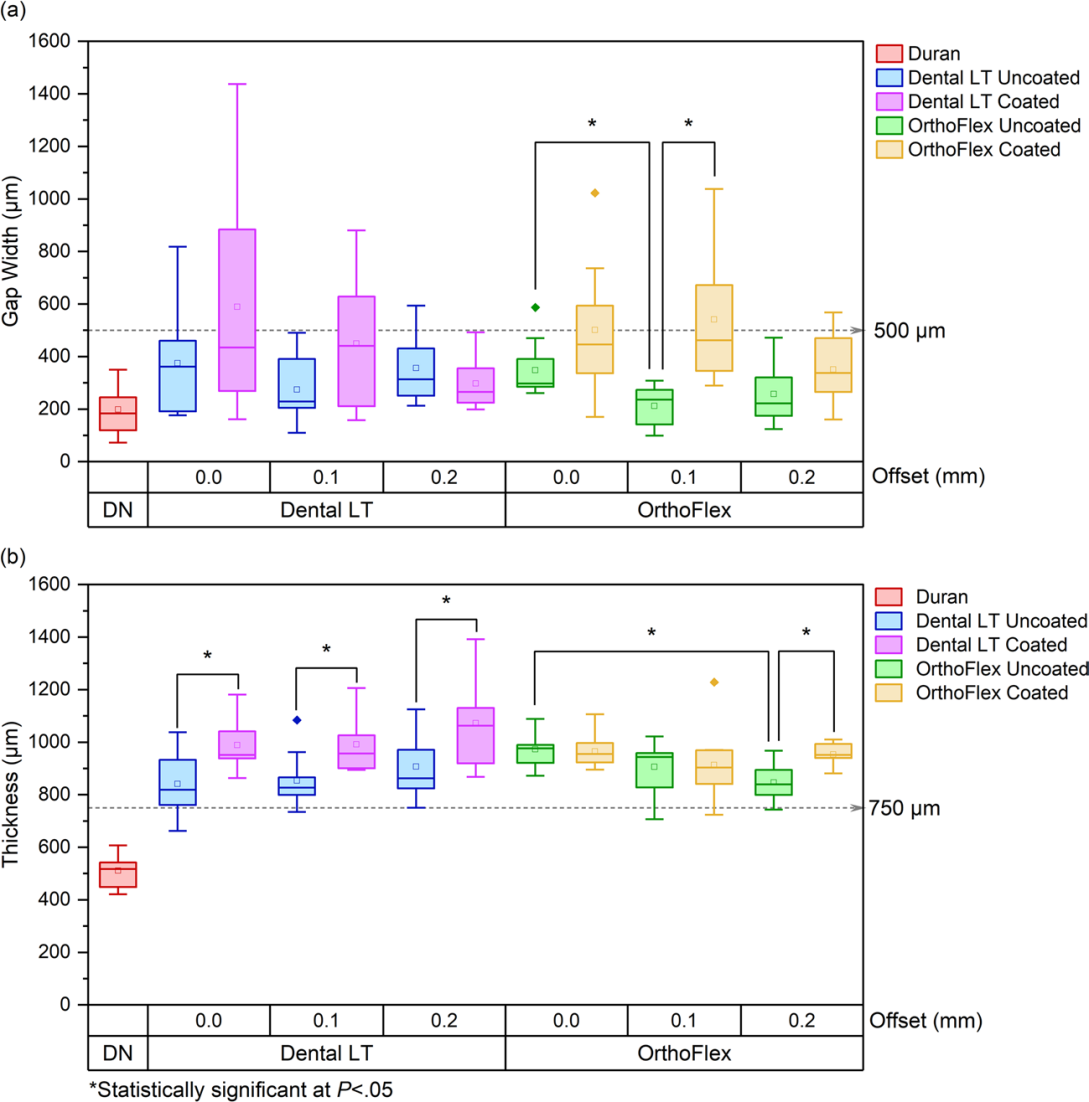
**a** Gap widths and **b** retainer thickness of each material and offset subgroups of the 3D-printing resins

For the gap width measurements, Duran had the narrowest average widths among all groups at 199.07 ± 87.53 μm. Different offsets did not produce statistically significant differences in gap width across all 3D-printing material subgroups, except in OrthoFlex Uncoated, where the gap width of 0 mm offset was significantly larger than of 0.1 mm offset. (Fig. 11a).

For the Dental LT material, the differences in gap width were neither significant between different offsets, nor at the same offsets between Dental LT Uncoated and Coated (*p* > 0.05). Dental LT Coated with 0 mm offset had the largest gap width of 588.94 ± 134.59 μm and the widest range from 161.32 to 1437.07 μm. The narrowest average gap widths in the Dental LT Uncoated group was for 0.1 mm offset at 274.54 ± 119.03 μm, while for the Dental LT Coated group was the 0.2 mm offset at 348.58 ± 105.89 μm. These two groups also did not have maximum gap widths above the threshold set at 500 μm.

Among the OrthoFlex subgroups, the highest average and maximum gap widths were seen in OrthoFlex Coated with 0.1 mm offset, at 541.60 ± 80.64 μm and 1037.94 μm respectively. The lowest was recorded for the same offset in OrthoFlex Uncoated, with an average gap width of 211.97 ± 78.00 μm. 0.2 mm offset produced the narrowest gap widths among the OrthoFlex Coated subgroups, with a mean of 350.65 ± 141.74 μm and range from 160.5 to 568.07 μm. Comparing between OrthoFlex Uncoated and Coated at the same offset, only the 0.1 mm offset produced a statistically significant increase in gap width (*p* < 0.05).

In terms of retainer thickness, Duran had the lowest mean at 510.89 ± 65.58 μm. All 3D-printed subgroups showed increased thickness above the original design of 750 μm regardless of offset distance. No statistically significant differences were seen between different offsets for the Dental LT material (*p* > 0.05), but coating significantly increased the thickness at all offsets (*p* < 0.05). For OrthoFlex Uncoated, the 0.2 mm offset had a significantly lower thickness than the 0 mm offset (*p* < 0.05), but no significant differences in thickness were detected between offsets for OrthoFlex Coated (*p* > 0.05). Coating only significantly increased the retainer thickness in the 0.2 mm offset group (*p* < 0.05) (Fig. 11b).

**Fig. 12:**
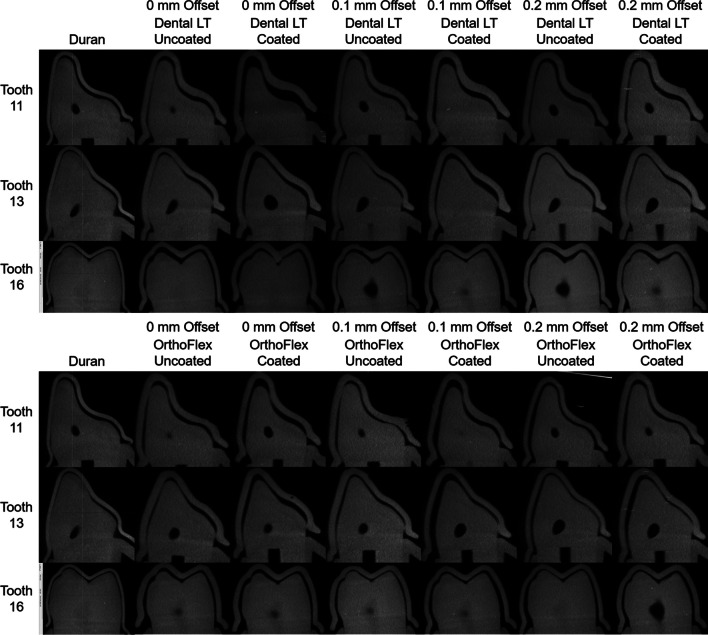
Cross-sectional micro-CT images of each group taken at the mid-buccopalatal plane of the maxillary right central incisor (tooth 11), canine (tooth 13), and first permanent molar (tooth 16)

From the cross-sectional micro-CT images (Fig. 12), the retainer samples fitted poorly in Dental LT Coated with 0 mm offset, and showed improved adaptation with increased offsets, especially at 0.2 mm offset. A similar trend could be seen in the OrthoFlex Coated subgroups. The Uncoated groups of both 3D-printing materials appear to fit and seat most properly using the 0.1 mm offset. Duran however, showed the closest adaptation to the tooth morphology and lowest thickness as compared to all 3D-printed subgroups.

## Discussion

It has been shown in our previous work that orthodontic clear retainers fabricated by direct 3D-printing have poor surface and optical characteristics which are directly related to the nature of the layered 3D-printing process [39]. As demand for orthodontic clear retainers has risen due to their aesthetic appearance, materials which are highly transparent and resistant to colour changes are desirable. Surface treatment is thus essential to improve on these aspects without jeopardizing the mechanical and dimensional properties of the 3D-printed clear retainers even after ageing.

All samples in this study were printed at a 45° angle in order to maximize the possible surface roughness created by the step edges of the 3D-printed layers [9, 40]. This would be able to demonstrate the extent of surface coating in reducing high roughness levels.

Surface roughness is often of much interest in the field of dental materials, especially those intended for intraoral use. The most common quantitative measure of surface roughness is the Ra or Sa value for 2D or 3D analysis, respectively, which is defined as the arithmetic mean departures of the surface profile from an average line derived from the top and bottom of the undulations on the trace [41].

Several thresholds of the Ra value have been reported and widely used in dentistry. The first is related to optical properties, where surfaces with roughness below 1 μm are considered reflective and impart a natural gloss [42]. The coated 3D-printed retainers in our study had Sa values below 1 μm and showed a level of gloss which was not present in the uncoated counterparts that had Sa values ranging from 3 to 13 μm. (Figs. 4 and 5) Another Ra threshold is concerned with clinical perceptibility or patient comfort. The surfaces of materials placed in the oral cavity are inevitably sensed by the patient’s tongue which has a high degree of sensitivity [43, 44]. Jones et al. proposed a sensitivity threshold of 0.5 μm [45], which is rational considering that the natural roughness of human enamel is 0.64 ± 0.25 μm [46]. Nevertheless, the Ra threshold that is most demanding is 0.2 μm, above which has been linked to levels of biofilm or microbial adhesion that could compromise oral hygiene [47].

Based on these three Ra threshold values, the Dental LT Coated group in our study could fulfil the first two limits, while OrthoFlex Coated would only be acceptable for the reflectivity threshold. As it was beyond the scope of our study, future research to determine whether the coating is able to adequately prevent microbial accumulation would be valuable.

The degree of transparency of a material can be explained by the interaction between the material with incident light. High levels of light transmission lead to transparency, while barriers to this produce opacity through reflective and refractive phenomena [48]. The multi-layered staircase surface structure inherent to the 3D-printing process would unavoidably cause light scattering at the air–solid interface, allowing limited wavelengths to pass through.

Visible light transmission of our samples increased from less than 10% to almost 80% when uncoated and coated, respectively, which can be associated with the decreased Sa values to below 1 μm for both 3D-printing materials. Park et al. and Kim et al. also showed improved transparency levels in 3D-printed clear aligners processed by centrifugation as opposed to IPA washing [20], and that these were comparable to the thermoplastic PET-G [17].

Although the resistance to staining has often been correlated to surface roughness, 3D-printed materials for dental applications tend to be more prone to colour changes as compared to conventional heat polymerized and milled materials regardless of post-processing protocol [40, 49–51]. Even though the Sa values of the coated 3D-printed materials in our study were comparable to the thermoplastic PET-G control, some groups especially OrthoFlex Coated after 12 days of immersion were still not as resistant to staining and even exceeded 12 NBS Units denoting ‘Very much’ change. In this case, the degree of polymerization, residual monomer leaching, water sorption, and type of photoinitiator instead of the surface roughness would be the underlying reasons for the poor colour stability of 3D-printed materials [52–55].

The type of staining solution is expected to affect each material differently [56]. All groups including the thermoplastic PET-G were highly susceptible to staining in curry even after only 24 h. The staining agent in curry is curcumin, a polyphenol found in the turmeric spice. Other studies have also observed high susceptibility of materials to curcumin staining [52, 57].

While the coated 3D-printing materials have displayed encouraging results in surface and optical properties, it would be important to ensure that the mechanical properties are not negatively affected either.

The distinct mechanical properties and resistance to ageing seen in our stress–strain curves can be related to the molecular structure of the materials, in particular cross-linking density, molecular weight, chemical composition, density, and crystallinity [58]. The high ductility of the thermoplastic material comes from the viscoelastic nature of long linear polymer chains, while methacrylate-based 3D-printing materials with a high density of cross-linking are unable to undergo a large magnitude of deformation before failure. The higher tensile strength of the dimethacrylate-based Dental LT resin also suggests that it has a higher molecular weight and density of cross-linking as compared to OrthoFlex.

As orthodontic retainers are exposed to the wet intraoral environment over a long time, water sorption and water solubility values could reflect the potential response of a material to thermal and hydrolytic degradation. Materials with low water sorption and water solubility values are desirable [59–61]. Water sorption is dependent on the chemical structure of the polymer and its interaction with water molecules, while water solubility is affected by the degree of polymerization of the polymer [62, 63]. The thermoplastic PET-G Duran had the lowest water sorption and solubility values (Table 5), and has thus shown to have high stability with minimal changes in surface composition after simulated ageing [37, 60].

For the 3D-printing materials, the water sorption was only marginally affected by the coating process as the chemical composition of the material was not altered. On the other hand, water solubility values dropped significantly in the coated groups, and its significance is reflected in their enhanced mechanical behaviour, which can be explained as follows: Firstly, the post-polymerization in an oxygen-free environment improves the degree of conversion of 3D-printed materials, increasing the material’s resistance to ageing by thermal and hydrolytic degradation [63–65]. Secondly, rinsing in organic solvents such as ethanol and IPA has a softening effect on composite resins as the solvents diffuse into the polymer matrix, causing swelling, dissolving linear polymer chains, and leaching of various components [66–68].

3D-printed objects in their freshly printed ‘green’ state are naturally covered with uncured liquid resin. Instead of rinsing off this uncured resin in an organic solvent, the excess is spun off to leave a thin uniform layer on the object surface perpendicular to the direction of the centrifugal force. As the coating material is essentially the same resin used to 3D-print the object, this removes the presence of a multi-material interface and its problems with bond failure. The final curing step would however need to be carried out in an oxygen-free environment to ensure that the polymerization of the uncured resin is not inhibited by atmospheric oxygen.

The thickness of film coatings depends on the properties of the coating solution such as density, viscosity, and surface tension, besides the rotation speed, duration, and radius [69]. Besides these factors, the printing technology can impact the behaviour of the residual resin film and resulting thickness of the coated samples. From Figs. 2 and 3, it is evident that the surface of the uncoated LCD-printed OrthoFlex material consisted of layers of high and low plateaus, giving rise to the highest maximum height, Sz measurements of around 65 to 77 μm. The uncoated SLA-printed Dental LT material had less prominent peaks and valleys, producing lower Sz values of about 30 to 40 μm. Despite these differences, their coated counterparts had Sz values that were statistically similar to one another as well as to the thermoplastic Duran. (Fig. 4) This infers that the uncured resin mainly filled the deeper undulations of the OrthoFlex surface, leading to less significant increases in overall appliance thickness. (Fig. 11b) On the other hand, the uncoated surface of Dental LT started out with relatively shallower peaks and valleys, so the uncured resin coat would have first filled the shallow voids, then overflowed and created a new surface film. Because of this, consistently significant increases in thickness of about 150–200 μm or more in the Dental LT samples for the same offset distance were detected, leading to larger gap widths throughout the retainer sample. (Fig. 11a).

For orthodontic retainers, a fitting accuracy threshold of 500 μm has been suggested as larger deviations are clinically significant as they put the teeth at risk of drifting into the space [70]. Our micro-CT results have shown that without a coating layer, an offset of 0.1 mm produced the narrowest gap widths of less than 500 μm for both Dental LT and OrthoFlex retainers, indicating clinically acceptable fit. A similar value was reported by Ye et al. using the IPA washing protocol with an unspecified resin for occlusal splints [71]. With coating however, an increased offset to 0.2 mm performed better than the original 0.1 mm offset. This can be associated with the changes in retainer thickness due to the coating layer as discussed previously. Hence, to account for the potential increase in thickness resulting from the coating layer, a sufficient offset of the internal fitting surface of 3D-printed orthodontic retainers should be incorporated into the 3D design.

To summarize our results, in order to ensure consistency in the properties of any appliances post-processed with a centrifuge protocol, the following parameters need to be clearly defined: 3D-printing technology, material type and viscosity, spinning speed, spinning duration, appliance size, appliance placement, and appliance design including internal offset. Further studies related to each of the factors are necessary to provide clinicians with reliable guidelines specific to each particular material and desired appliance.

Our study has highlighted the benefits of addressing the compromised surface properties of 3D-printed orthodontic clear retainers, especially in terms of optical properties without being detrimental to their mechanical properties. The post-processing procedure has clearly affected the properties of 3D-printing resins. Although the coating material in our study is essentially the same 3D-printing resin, it was outside the scope of this study to determine whether the biological response in terms of cytotoxicity and microbial adhesion, besides the leaching of monomers would be negatively affected. Therefore, this or any experimental protocol that alters the properties of the final product should always be taken with caution as it deviates from the manufacturer’s recommendations [72].

In addition, the sample size for each test in this study was relatively small and could be improved to increase the accuracy of data analysis, but we believe that our results have provided a big-picture overview of the main properties concerning orthodontic clear retainers.

## Conclusions

Post-processing by centrifugation instead of IPA cleaning improved the surface roughness and thus the transparency and colour stability of 3D-printed orthodontic clear retainers. The extent of improvement in the surface and optical properties mainly depends on the 3D-printing technology.

Post-curing in an oxygen-free environment enhances the mechanical properties and resistance to ageing, which in turn depend upon the chemical composition and structure of the 3D-printing material.

The increase in thickness resulting from the centrifuge coating protocol is related to the type of 3D-printing technology and would thus require designing an additional offset to ensure proper fit of the appliance.

## Data Availability

No datasets were generated or analysed during the current study.
